# Elimination of Intravenous Di-2-Ethylhexyl Phthalate Exposure Abrogates Most Neonatal Hypertension in Premature Infants with Bronchopulmonary Dysplasia

**DOI:** 10.3390/toxics9040075

**Published:** 2021-04-02

**Authors:** Randall Jenkins, Katia Farnbach, Sandra Iragorri

**Affiliations:** Department of Pediatrics, Oregon Health & Science University, Portland, OR 97239, USA; Farnbach@ohsu.edu (K.F.); Iragorri@ohsu.edu (S.I.)

**Keywords:** hypertension, prematurity, phthalates, di-2-ethylhexyl phthalate (DEHP), bronchopulmonary dysplasia, epigenetics

## Abstract

(1) Background: The incidence of hypertension in very low birthweight (VLBW) infants in a single neonatal intensive care unit (NICU) dropped markedly during a 2-year period when the IV fluid (IVF) in both the antenatal unit and the NICU temporarily changed to a di-2-ethylhexyl phthalate (DEHP)-free formulation. The objective of the current report is to document this observation and demonstrate the changes in incidence of hypertension were not associated with the variation in risk factors for hypertension; (2) Methods: The charts of all VLBW infants born in a single NICU during a 7-year span were reviewed. This time includes 32 months of baseline, 20 months of DEHP-free IVF, 20 months of IVF DEHP re-exposure, and two 4-month washout intervals. The group of interest was limited to VLBW infants with bronchopulmonary dysplasia (BPD). Chi-square analysis was used to compare incidence of hypertension among periods. Vermont Oxford NICU Registry data were examined for variation in maternal and neonatal risk factors for hypertension; Results: Incidence of hypertension in VLBW infants with BPD decreased from 7.7% (baseline) to 1.4% when IVF was DEHP-free, rising back to 10.1% when DEHP-containing IVF returned to use. Risk factors for neonatal hypertension were stable across the 3 study periods in the NICU’s group of VLBW infants; (3) Conclusions: Serendipitous removal of IVF containing DEHP resulted in near elimination of hypertension in one NICU—an effect entirely reversed after the same brand of DEHP-containing IVF returned to clinical use. These results suggest that DEHP exposure from IVF plays a major role in neonatal hypertension.

## 1. Introduction

Phthalates are a group of synthetic industrial chemical compounds used in the manufacturing of toys, cosmetics, pharmaceuticals, fragrances, baby-care products, food packaging, and medical supplies [1,2,3,4]. Di-2-ethylhexyl phthalate (DEHP) remains the only phthalate approved for use in medical supplies by the FDA. More than two million tons of DEHP per year are produced worldwide [5]. When added to polyvinyl chloride (PVC) DEHP makes devices softer and more flexible [6].

Phthalate toxicity research first focused on cancer risk, followed by reproductive and developmental risks [7]. Given differences between animal and human metabolism, the risk to humans remained controversial through 2003 [7,8]. Attention then turned to phthalate risks to small infants, as many studies identified alarming amounts of DEHP leaching from medical plastics into infants [9,10,11,12,13,14,15,16,17], yet the demonstration of actual toxicity in these infants remained scant [18]. Shiue et al. reported in 2012 an association between urinary phthalate levels and high blood pressure in adults [19], followed quickly by similar reports in children by Trasande et al. [20,21]. Flynn et al. also raised awareness of the possible cardiovascular risks from phthalates in their 2017 guidelines [22].

In 2019, our group reported an association between phthalate exposure in premature infants and increased systolic blood pressure (SBP) and hypertension (HTN) [23]. DEHP exposures were identified in intravenous (IV) fluids, IV tubing, and respiratory devices. We found a linear relationship between cumulative IV DEHP exposure and SBP measured at 40 weeks postmenstrual age (PMA). Statistical analysis showed this relationship was mediated by the cortisol-to-cortisone ratio, suggesting that the DEHP effect on blood pressure was due to activation of the mineralocorticoid receptor via inhibition of 11β-HSD2.

Despite this, the relationship between IV DEHP and hypertension was confounded by chronic lung disease (CLD), low birthweight, and prolonged length of stay in the neonatal intensive care unit (NICU) [23]. Other risk factors of non-secondary neonatal hypertension include, maternal hypertension, and antenatal steroid exposure [24,25,26,27,28]. Reports show that the majority of unexplained (non-secondary) neonatal hypertension cases have bronchopulmonary dysplasia (BPD) the type of CLD seen in premature infants [29,30,31]. Still, there is no consensus as to the pathophysiology behind the association of BPD and neonatal hypertension [32,33,34].

During a hypertension surveillance, we became aware of a seemingly unexplained, abrupt, and lasting decrease in the incidence of HTN in one NICU, only to return to prior levels after 2 years. On further analysis we discovered conventional IV fluid changed (both in the NICU and the antenatal wards) from a brand containing DEHP to a brand labeled DEHP-free [29]. The original DEHP containing IVF returned to clinical use after a 2-year hiatus period. These serendipitous changes in IV DEHP exposure allowed us to evaluate the effect of IV DEHP without the need for a controlled trial, and to address the effect of BPD and other confounding associations on the genesis of neonatal hypertension.

## 2. Materials and Methods

This work is part of an institutionally approved quality initiative, tracking incidence of neonatal hypertension in one neonatal unit over time. Cases of HTN were identified both by logs of nephrology referrals for hypertension and by querying the electronic record for patients with hypertension in the problem list or discharge diagnosis. In lieu of a specific control group, we used the Vermont Oxford Registry to examine the unit-specific demographic and hypertension risk factors.

The review was limited to premature very low birth weight (VLBW) infants, as this is the population at highest risk for hypertension [29]. This project was reviewed by the institutional research committee who classified it as a quality improvement project, for which no informed consent was required. Inclusion criteria were prematurity (born at <37 weeks’ gestation), a birthweight between 500 and 1500 g, diagnoses of both hypertension and BPD, and having a birthdate between 1 January 2014 and 31 August 2020. The criterion for the diagnosis of hypertension was three days or more where the average of three or more systolic blood pressure (SBP) readings was greater than the 95th percentile for postmenstrual age (PMA) as defined by Dionne [35]. The diagnosis of BPD was based on either a continuing need for respiratory support after 36 weeks PMA, or oxygen for more than 28 days from birth. We excluded patients with secondary hypertension based on a list developed by Flynn in 2012 [36], using the descriptive criteria as published by Farnbach et al. in 2019 [29] (Table 1).

The study timeline includes a 32-month baseline period (Period 1), a 20-month IV DEHP-free period (Period 2), and a 20-month period (Period 3) during which the same brand of DEHP-labeled IV fluid used in Period 1 returned to clinical use. The timeline excluded four-month blocks during each changeover to eliminate uncertainty as to which supplies were actually being used. IV fluid for this study refers only to the commercially available IV fluid used both in the NICU and in the antenatal wards including bags of normal saline, ringer’s lactate, and dextrose in water. It does not include IV fluid mixed with medication, nor bags containing total parenteral nutrition (TPN) which did not change during the study.

### Study Procedures

Incidence of HTN in BPD patients was calculated for each period based on the number of new HTN cases per total number of VLBW premature infants born at or transferred into one tertiary NICU. We compared the incidence of HTN between the periods with and without DEHP exposure in IV fluids. In addition, we searched for variations in birthweight or gestational age, as well as other risk factors of neonatal hypertension that could have explained the observed changes in hypertension incidence. Analyzed maternal risk factors included frequency of maternal hypertension and antenatal steroid use. Neonatal risk factors included, chronic lung disease (CLD) incidence, and length of stay (LOS). Unit-specific risk factor data for VLBW infants were obtained from the Vermont Oxford Registry database. Chi-square testing was used to compare nominal data between periods. Student’s *t* testing was used to compare continuous data among periods.

## 3. Results

We identified a total of 49 cases of hypertension in a single NICU over the three time periods. Thirty-five of those were in VLBW infants. The distribution of these 35 cases in relation to the periods of DEHP exposure, withdrawal and re-exposure is shown in Table 2. Thirty-one of 35 VLBW infants (89%) had both hypertension and BPD—the interest subgroup. Seventeen of 31 were born during the initial 32-month baseline period, 2 of 31 were born during the 20-month IV DEHP-free period, and 12 of 31 were born during the 20-month period when DEHP-labeled IV fluid was reintroduced. Clinical characteristics of the VLBW hypertensive patients with BPD from each period are shown in Table 3.

Table 2 demonstrates how the incidence of HTN in the VLBW infants with BPD dropped dramatically from 7.7% at baseline, to 1.4% during the DEHP-free IV fluid period; only to bounce back up to 10.1% during period 3 when DEHP-labeled IV fluids were reintroduced. Table 4 demonstrates no significant differences in demographics including length of stay across the periods. Of risk factors for HTN, only antenatal steroids administration was higher in period 3 versus period 2, albeit not different when comparing period 1 with period 2, or the combined DEHP-positive periods (1 & 3) versus period 2. Other risk factors were not different among the three periods.

## 4. Discussion

Upon the removal and return of IV DEHP, dramatic changes in the incidence of “BPD-associated” HTN suggest IV DEHP may play a major role in the genesis of hypertension in VLBW infants. Historically hypertension in this patient population has been attributed to BPD. Yet, during this surveillance, the changes in hypertension incidence were not mirrored by changes in unit-specific CLD incidence. From this, we must conclude the association between BPD and hypertension in VLBW infants appears not to be a causal one. We have found no other plausible reason to explain these dramatic fluctuations in the incidence of hypertension across the study periods and we are confident we did not miss detecting cases of hypertension during Period 2, when the IV fluids were DEHP-free.

The mechanism by which IV phthalates might cause HTN is inconclusively understood. Zhao previously had shown that monoester metabolites of DEHP inhibit 11β-HSD2 in human microsomes [37]. This effect resembles that described with the ingestion of black licorice and has the same pathophysiology of apparent mineralocorticoid excess [38]. This pathophysiologic mechanism was verified in a small group of premature infants when we demonstrated: (1) A linear relationship between IV DEHP and systolic blood pressure index with cortisol-to-cortisone ratio, a surrogate for 11β-HSD2, mediating that effect; (2) Elevated DEHP urine metabolites in DEHP-exposed infants; (3) Increased sodium channel markers’ (ENaC and pNCC) expression in hypertensive compared with normotensive infants [23].

There are still many unanswered questions concerning the relationship between IV DEHP and neonatal hypertension. Not least the one that addresses the delay between IV DEHP exposures (which usually occurs during the first few weeks of life) and the onset of HTN (which typically commences towards 40 weeks PMA). This gap of time raises at least 2 possibilities. One refers to the required maturation of the mineralocorticoid receptor-controlled sodium channel expression. Our recent report in premature infants showed that sodium channel expression in normotensive or hypertensive infants was minimal until at least 34 weeks PMA [23]. The other possibility is the potential for epigenetic change caused by maternal (or early postnatal) exposure to DEHP through IV fluid. Epigenetic changes are well recognized effects of phthalates [39,40].

The major strength of this work is the serendipitous occurrence of the classic withdrawal and re-challenge “experiment” for the use of IV fluids containing DEHP. Our study’s weakness is that it is underpowered to detect any impact IV DEHP may have on less frequent categories of neonatal HTN.

## 5. Conclusions

IV DEHP exposure appears to play a role in the origin of hypertension in VLBW infants in the NICU. BPD does not appear to be causal of neonatal hypertension. The role of maternal DEHP exposure, including the possibility of an epigenetic effect is still not clear but intriguing. The relative role of other sources of neonatal phthalate exposures in the genesis of neonatal hypertension is also unknown—for example, exposures from respiratory devices. Further study is necessary to help us better understand the effect these might have on the incidence of HTN in premature infants.

## Figures and Tables

**Table 1:** Categories of neonatal hypertension from Flynn et al. with criteria for category placement. Reproduced from [29], 2019, Nature.

Categories	Criteria
Renovascular	Renal artery thromboembolism or renal vein thrombosis
Congenital renal parenchymal disease	Significant renal parenchymal or urological congenital anomalies, including moderate or severe hydronephrosis, cystic dysplasia, or renal hypoplasia
Acquired renal parenchymal disease	Serum creatinine greater than 0.6 mg/dL or urine output <1 mL/kg/h at the time of hypertension and without other mechanism of hypertension
Pulmonary	Chronic lung disease (respiratory support needed after 36 weeks postmenstrual age or 28 days of chronologic age) or acute pulmonary disease at hypertension onset
Cardiac	Coarctation of the aorta, supra-valvular aortic stenosis, or mid-aortic syndrome
Endocrine	Hyperthyroidism, congenital adrenal hyperplasia, Cushing’s syndrome, Cohn’s syndrome, or any monogenic cause of hypertension
Medications/Intoxicants	Patients exposed to one or more of the following agents: corticosteroids, ACTH, sympathomimetics, stimulants, fluid overload, or excessive sodium administration
Neoplasia	Wilms tumor, neuroblastoma, pheochromocytoma, or mesoblastic nephroma
Neurologic	Increased intracranial pressure or intraventricular hemorrhage at the time of hypertension
Miscellaneous	Patients not fitting other categories or for whom a secondary cause of hypertension could not be identified

**Table 2:** Distribution of cases of hypertension in very low birthweight infants across periods of varying intravenous DEHP exposure.

Categories of Hypertension ^a^	Period 1 32 Months *Baseline*	Period 2 20 Months *Removal*	Period 3 20 Months *Return*	*p*	
	IV DEHP+ 221 VLBW	IV DEHP− 144 VLBW	IV DEHP+ 119 VLBW	Period 1 vs. 2	Period 2 vs. 3
	*n* (%)	*n* (%)	*n* (%)		
Pulmonary	18 (8.1)	2 (1.4)	12 (10.1)	<0.01	<0.01
Bronchopulmonary dysplasia	17 (7.7)	2 (1.4)	12 (10.1)	<0.01	<0.01
Acquired renal parenchymal disease	1 (0.5)	0 (0)	0 (0)	NS	NS
Miscellaneous	3 (1.4)	0 (0)	0 (0)	NS	NS
All-cause hypertension	21 (9.5)	2 (1.4)	12 (10.1)	<0.01	<0.01

^a^ No cases of hypertension due to renovascular, congenital renal parenchymal disease, cardiac, endocrine, medications/intoxicants, neoplasia, or neurologic categories were identified during the study period. DEHP, di-(2-ethylhexyl) phthalate; VLBW, very low birthweight; NS, not significant using *p* < 0.05 for significant difference.

**Table 3:** Demographics and risk factors of neonatal hypertension in 31 VLBW infants with BPD over time.

Risk Factors	Period 1 2014–2016 *n* = 17	Period 2 2017–2018 *n* = 2	Period 3 2019–2020 *n* = 12
Male gender (%)	76	0	50
Birthweight (kg)	1.1 ± 0.2	0.9 ± 0.1	0.9 ± 0.2
GA at birth (weeks)	27.8 ± 1.6	29.5 ± 4.0	27.2 ± 1.7
PMA at HTN (weeks)	39.4 ± 2.5	40.1 ± 3.6	39.4 ± 2.2
Bronchopulmonary dysplasia (%)	100	100	100
Incidence of maternal hypertension (%)	23.5	0	30.0

VBLBW, Very low birthweight (500–1500 g); BPD, Bronchopulmonary dysplasia; GA, Gestational age; HTN, Hypertension; PMA, Postmenstrual age.

**Table 4:** Demographics and hypertension risk factors from unit-specific registry data in all VLBW infants.

Demographics and Risk Factors	Period 1 2014–2016	Period 2 2017–2018	Period 3 2019–2020	Period 1 + 3	*p*		
	*n* = 267	*n* = 191	*n* = 98	*n* = 365	Period 1 vs. 2	Period 2 vs. 3	Period 1 + 3 vs. 2
Birthweight (kg) ^+^	1.1 ± 0.3	1.0 ± 0.3	1.1 ± 0.3	1.1 ± 0.3	NS	NS	NS
GA at birth (weeks) ^+^	28.5 ± 3.1	28.1 ± 3.2	28.3 ± 2.8	28.5 ± 3.0	NS	NS	NS
Length of stay (days) ^+^	59.1 ± 40.1	56.0 ± 38.8	62.5 ± 41.0	60.0 ± 40.3	NS	NS	NS
Antenatal steroids (%)	82.5	82.2	94.9	85.8	NS	0.003	NS
Chronic lung disease (%)	25.4	27.4	35.6	28.9	NS	NS	NS
Incidence of maternal hypertension (%)	27.2	28.8	33.7	29.0	NS	NS	NS

^+^ Continuous variables are expressed as mean ± SD. VLBW, very low birthweight; GA, gestational age; NS, not significant using *p* < 0.05 for significant difference.

## Data Availability

Data sharing not applicable. No new data were created or analyzed in this study other than what appears.

## Glossary

DEHP	Di-2-ethylhexyl phthalate
VLVW	Very low birthweight (birthweight of 500–1500 g)
NICU	Newborn intensive care
IVF	Intravenous fluid
BPD	Bronchopulmonary dysplasia
FDA	Food and Drug Administration
SBP	Systolic blood pressure
HTN	Hypertension
PMA	Postmenstrual age
11B-HSD2	11 beta-hydroxysteroid dehydrogenase type II
CLD	Chronic lung disease
TPN	Total parenteral nutrition
LOS	Length of stay
GA	Gestational age
